# Effects of heat reflux extraction on the content, antioxidant, and immune activity of polyphenols and flavonoids from hempseed threshing residues

**DOI:** 10.1371/journal.pone.0322134

**Published:** 2025-05-07

**Authors:** Xiao-Jun Li, Juan Guo, Yong-Ping Xu, Shu-Ying Li, Na Li, Qing-Ye Liu

**Affiliations:** 1 School of Chemistry and Chemical Engineering, North University of China, Taiyuan, China; 2 School of Bioengineering, Dalian University of Technology, Dalian, China; 3 SEM Bio-Engineering Technology Co. Ltd., Dalian, China; 4 School of Biological Science and Technology, Taiyuan Normal Univercity, Jinzhong, China; Korea University - Sejong Campus, KOREA, REPUBLIC OF

## Abstract

**Objective:**

Hempseed threshing residues are rich in phytochemicals such as polyphenols and flavonoids. Phenolic and flavonoid compounds have been associated with antioxidant, antibacterial and anticancer activities. The re-use of the hempseed threshing residues as value-added materials is, not only cost-saving, but also environmentally beneficial. It is therefore important to develop an effective method for extraction of phenolic compounds and flavonoids from hempseed threshing residues.

**Methods:**

In this investigation, the extraction of phenolic constituents and flavonoids from hempseed threshing residues using heat reflux extraction (HRE) were optimized through response surface methodology (RSM). Four HRE parameters to enhance the yield of crude extracts (CE), total phenolic content (TPC), and total flavonoids content (TFC) were evaluated. Additionally, the study evaluated the chemical compounds, antioxidant characteristics of the extracts, and the immune activity of the extracts was assessed by quantifying the levels of inflammatory cytokines, specifically IL-6, IL-10, and TNF-α.

**Results:**

The best extraction parameters were determined as: for the extraction time of 69.71 min, a liquid-solid proportion of 5.12:1, a particle size of 1150 µm, and an ethanol concentration of 69.60%. Under these optimized conditions, the yields for CE, TPC, and TFC were 4.74%, 27.54%, and 16.02% respectively. The data conformed well to multiple regression models, showing that these extraction parameters markedly influence the yields of CE, TPC, and TFC. Most of the compounds found may belong to the class of polyphenol and flavonoids. Cellular assays indicated that extracts from hempseed threshing residue notably reduced pro-inflammatory factors (TNF-α, IL-6) and increased anti-inflammatory factors (IL-10) in RAW 264.7 cells.

**Conclusion:**

This research lays a theoretical foundation for extracting polyphenols and flavonoids from hempseed threshing residue and for the comprehensive assessment of antioxidant and immune-enhancing products. However, the antioxidant and immune activity of hempseed threshing residues extracts under physiological conditions in vivo, and the relevant mechanism should be further studied.

## Introduction

Hemp (*Cannabis sativa*), potentially originating from China, is classified as a C_3_ plant. Historical records suggest hemp cultivation dating as far back as 2700 BC [1] Industrial hemp, known for its minimal △^9^-tetrahydrocannabinol (△^9^-THC) content, the component linked to psychotropic effects [2], has been selectively bred for its rich oil and protein content in seeds or for its high fiber yield in stems [3]. Hemp fibers are utilized in textile and paper industries. The seeds produce an oil devoid of △^9^-THC, abundant in linoleic and α-linolenic fatty acids [4], making it suitable for functional foods and cosmetic production [5]. The introduction of a threshing process in hempseed processing has enhanced oil quality but also increased waste, such as hempseed hulls. Addressing the challenge of harmless and value-added utilization of hempseed threshing residues is crucial for the industry. Repurposing these residues not only saves costs but also benefits the environment. Research indicates that these residues are rich in phytochemicals like polyphenols and flavonoids [6,7], which are identified for their antioxidant, antibacterial, and anticancer properties [8]. Therefore, developing an efficient technology for extracting these compounds from hempseed threshing residues is of significant importance.

Various extraction technologies are crucial for isolating active ingredients from plant matrices. These methods include cold dip extraction, heat reflux extraction (HRE), ultrasound or microwave-enhanced extraction, and extraction using supercritical fluids [9–11]. Among these, HRE is particularly prevalent due to its distinct advantages. This method enhances the temperature of solvent, significantly speeding up the diffusion of active components and boosting extraction efficiency, Besides, the HRE can process more samples at the same time and carry out batch operation, which is suitable for industrial-scale production [12,13]. However, there are some potential drawbacks such as high energy consumption, or degradation of sensitive compounds during prolonged heating. Therefore, it is necessary to optimize the heat reflux extraction process. There are many factors that influence extraction efficiency, such as liquid-solid proportion, particle size, extraction duration, and ethanol content. The influence of solid-liquid ratio and particle size on extraction efficiency is mainly reflected in the contact area and concentration gradient between solvent and raw material. Suitable ethanol concentration and extraction duration can maximize the leaching amount of the target substance, but there has been no reported use of HRE for extracting total polyphenols and flavonoids from hempseed threshing residues, presenting a novel area for investigation.

This study focused on examining the key factors affecting the heat reflux extraction of polyphenols and flavonoids substances from hempseed threshing residues. The aim was to enhance the extraction procedure utilizing the BBD-RSM. Additionally, the study evaluated the antioxidant characteristics of the extracts, employing tests like DPPH free radical scavenging, ·HO scavenging, diminishing power, and FRAP assays. Moreover, the immune activity of the extracts was assessed by quantifying the quantities of inflammatory cytokines, specifically IL-6, IL-10, and TNF-α.

## Materials and methods

### Materials and reagents

In October 2022, hempseed threshing residues were obtained from Shanxi Hongtian Jiali Agricultural Technology Co., Ltd., located in Jinzhong, Shanxi Province, China. These residues underwent air drying and subsequently stored at -18°C to preserve their quality.

DPPH, TPTZ, FC reagent, gallic acid, rutin, and Vc were sourced from Sigma Chemical Co. in St. Louis, Missouri, USA. Additionally, various laboratory grade reagents including ferrous sulfate, sodium nitrite, aluminum chloride, sodium dihydrogen phosphate, glacial acetic acid, hydrogen peroxide, potassium ferricyanide, sea salt, and ethanol were procured from Tianjin Beichen District Fangzheng Reagent Factory, located in Tianjin, China.

### Extraction and determination of TPC and TFC

In this study, the conventional HRE method, commonly utilized in industrial applications, was employed. Initially, 200 grams of powdered hemp seed threshing residues (with a particle size of 850 μm) underwent extraction twice using 70% (v/v) ethanol. This process was conducted in a 2-liter flask for 60 minutes, maintaining a liquid-solid proportion of 5:1 (mL/g). Subsequently, the mixture was concentrated under reduced pressure to remove ethanol and then centrifuged at 5000 × g for 15 minutes, to separate the super-natant. The collected super-natant was then subjected to spray drying using an ER-1500 from Yiyan, Shanghai, China, at 40°C, a feed flow rate of 15 mL/min, and an input air temperature of 180–190°C. The resulting extract powder was kept at -18°C until subsequent examination. The output of the CE% was computed using a specific formula (1). TPC was confirmed with the Folin-Ciocalteu technique and presented as a percentage of GAE% [14]. TFC was ascertained using the aluminum chloride colorimetric technique, with rutin as the benchmark, and the findings were reported as a percentage of rutin equivalent in the crude extract (RE%) [15].


CE( % )=weight of crude extract (g)/weight of hemp seed threshing residues (g) ×  %  (1)

### Single-factor experiments

The extraction of polyphenols and flavonoids from hempseed threshing residues was carried out using a heat reflux system in a condensation setup, following a single-factor experimental design. This design incorporated four key variables: liquid-solid proportion, particle size, extraction duration, and ethanol content. Specifically, liquid-solid proportions tested were 3:1, 4:1, 5:1, 6:1, and 7:1, using hempseed residues with a particle size of 850 μm and extracting with 70% ethanol for 60 minutes, repeated twice. Extraction times evaluated were 30, 60, 90, 120, and 150 minutes, maintaining a liquid-solid proportion of 5:1 and using 70% ethanol, also repeated twice. The particle sizes examined ranged from 2000, 850, 420, 250, to 180 μm, each extracted with 70% ethanol at a liquid-solid proportion of 5:1 for 60 minutes, and performed twice. Finally, ethanol concentrations of 50%, 60%, 70%, 80%, and 90% were tested, extracting hempseed residues of 850 μm particle size by heat reflux for 60 minutes with a liquid-solid proportion of 5:1, and this procedure was repeated twice as well.

### RSM experimental design

The RSM was employed to enhance the heat reflux extraction for complete polyphenols and flavonoids from hempseed threshing residues. In this model, four distinct factors were selected: liquid-solid proportion (X_1_), extraction duration (X_2_), particle size (X_3_), and ethanol concentration (X_4_). These variables were varied across three levels: high (1), mid (0), and low (−1). TPC and TFC were chosen as the outcome factors (Y). The RSM was structured to include 29 experimental runs, accommodating three levels for each of the four variables.

### Analysis of chemical compounds by high-performance liquid chromatography-mass spectrometry (HPLC-MS)

The chemical compounds of extract were detected by H-Class HPLC (Waters, Milford, USA) with Waters BEH C18 chromatography column (2.1 × 50 mm, 1.7μm), and analyzed by Xevo G2-XS Qtof MS (Waters, Milford, USA). The mobile phase was 0.1% formic acid in 90% methyl alcohol at a flow rate of 0.6 mL/min. The column temperature was 40°C, and the sample injection volume was 10 μL. The normal negative-mode ESI-MS conditions were as follows: Spray gas pressure of 50 psig, dry gas flow rate of 800 L·h^-1^, drying temperature of 400°C, capillary voltage of 3500 V, ion source temperature of 110°C, and scanning range (m/z) of 50 ~ 2000.

### Assessment of in vitro antioxidant activity

The DPPH· scavenging efficacy was evaluated utilizing an altered version of the procedure outlined by Choi [16] *et al*. Concentrations of hempseed threshing residue extract were prepared via serial dilution in ethanol. The process involved mixing 0.3 mL of the diluted extract with 2.7 mL of a 60 μM DPPH solution in ethanol. This combination was subsequently permitted to undergo a reaction at 25°C for 30 min protected from light. The absorbance at 517 nm was quantified utilizing UV-visible spectroscopy. Vc acted as the affirmative reference in this experiment. The scavenging capacity of the extract samples was determined through a specific formula designed for this purpose:


$$\begin{array}{c} \text{DPPH scavenging activity ( }\;\;\%\;\;\text{ )}=\;\;\;[\;\;\text{ (A\textbackslash{}textsubscript\{control}\}-\text{A\textbackslash{}textsubscript\{sample})/A\mathrm{\backslash textsubscript}control\}\;\;]\;\;\;\times \text{100} \end{array}$$ (2)

The ·HO scavenging assay was performed in accordance with the technique characterized by de Avellar [17] *et al.* Various concentrations of hempseed threshing residue extract and Vitamin C Vc were prepared using serial dilution in water. Both 1,10-phenanthroline (0.5 mM) and FeSO_4_ (0.75 mM) were solubilized within a 0.2 M phosphate buffer at pH 7.4. The response was commenced by introducing 0.01% H_2_O_2_ and then kept at 37°C for 60 min. Subsequently, the absorbance of the outcome blend was gauged at 536 nm. Absorbance readings for a blank solution (lacking both the sample and H_2_O_2_ and a control (without the sample) were also recorded. The**·**HO scavenging capability of the extract specimens was calculated using a specific formula designed for this assay:


$$\text{HO scavenging activity }\left(\;\;\;\%\;\;\;\right)\text{ = }\left[\left({\text{A}}_{\text{sample}}\text{- }{\text{A}}_{\text{control}}\right)\text{/}\left({\text{A}}_{\text{blank}}\text{- }{\text{A}}_{\text{control}}\right)\right]\;\;\;\times \;\;\text{ 100}$$ (3)

The reducing power assay was conducted based on modified procedures outlined by Arts [18]. For this assay, 1 mL of the test sample solution was combined with 2.5 mL of phosphate buffer (0.2 M, pH 7.0) and 2.5 mL of potassium ferricyanide (1%, w/v). The blend was initially kept at 50°C for 20 min. After this cultivation duration, 2.5 mL of trichloroacetic acid (10%, w/v) was introduced, and the blend was subsequently centrifuged at 3500 g for 10 minutes. After centrifugation, 2.5 mL of the super-natant was combined with 0.5 mL of newly made ferric chloride solution (0.1%, w/v). The absorbance of this ultimate blend was assessed at 700 nm after resting for 10 minutes. Vc was utilized as the positive reference in this assay. Additionally, a blank sample, which did not include the test compound, was prepared for comparison.


$$\text{Increase in reducing power }\left(\;\;\;\%\;\;\;\right)\text{ = }\left[\left({\text{A}}_{\text{sample}}\text{-- }{\text{A}}_{\text{blank}}\right)\text{/}{\text{A}}_{\text{blank}}\right]\;\;\;\times \;\;\text{ 100}$$ (4)

The FRAP assay was conducted with certain modifications to the methodology initially outlined by Benzie [19] *et al.* In this modified assay, 0.3 mL of the experimental specimen mixture was combined with 4.5 mL of freshly prepared FRAP reagent. The FRAP reagent was composed of 50 mL of 0.1 M acetate buffer solution (pH 3.6), 5 mL of 20 mM FeCl_3_, and 5 mL of a 10 mM TPTZ solution in 40 mM HCl. This combination was then maintained at 37°C for 30 min. Following incubation, the absorbance reading of the solution was gauged at 593 nm, employing a reagent blank as the reference. The outcomes of the test were reported with regard to μmol Vc per gram weight of the crude extract.

### Immune activity

#### Toxicity of extracts on RAW264.7.

RAW264.7 cells were grown in DMEM complete medium enhanced with 10% FBS and kept at 37°C within a specific environmental setting comprising 5% CO_2_. These cells, with a concentration of 5 × 10^4^ cells per well, were placed in 96-well plates and permitted to cultivate for 1 day. The toxic effects of the specimens on RAW 264.7 cells were evaluated through the MTT assay approach, as outlined by Li [20] *et al.*

#### Determination of IL-6, IL-10 and TNF-α.

RAW 264.7 cells were cultured in DMEM complete medium supplemented with 2 μg/mL LPS for a period of 24 hours. Post this incubation, the medium was discarded, and solutions of the extracts at levels of 100, 200, and 400 μg/mL were introduced to the cells in a 96-well plate. For control purposes, wells with no extracts (serving as a blank control) and wells without LPS (acting as a positive control) were also prepared. After an additional 24 hours of incubation, the culture super-natant was collected. The concentrations of IL-6, IL-10, and TNF-α in the super-natant were then quantified according to the protocols provided with the IL-6, IL-10, and TNF-α immune response kits from Beyotime Biotechnology, Jiangsu, China.

### Statistical analysis

All information in the research were reported as the mean ± SD and were examined utilizing SPSS version 22.0, a statistical software provided by IBM Corporation, based in Armonk, NY, USA.

## Results and discussion

### Single-factor study

**Fig 1:**
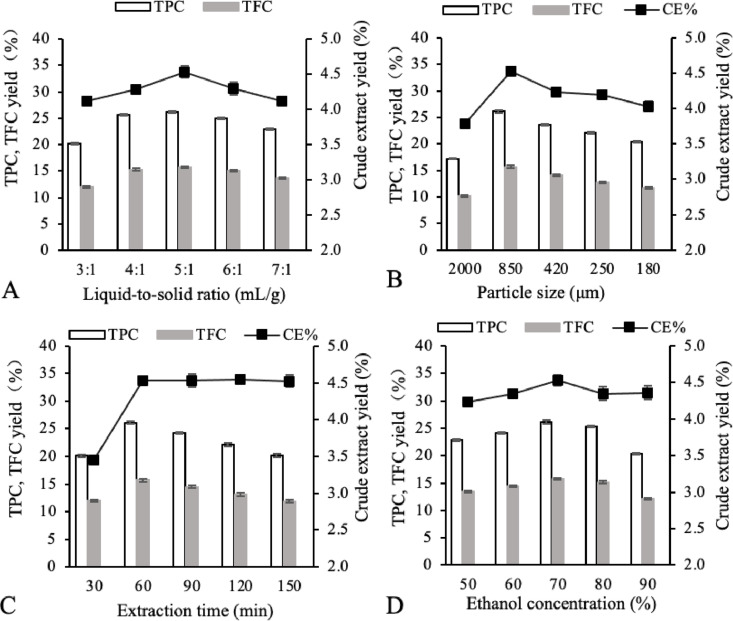
The effect of liquid-solid proportion. (A), particle size (B), extraction time (C) and ethanol concentration(D) on the extraction yields of CE, TPC and TFC.

Fig 1(A) depicts the consequences of the liquid-solid proportion on the output of the extraction of crude extract, polyphenols, and flavonoids. The optimal ratio for these extractions was found to be 5:1 (mL/g). It was observed that increasing the liquid-solid proportion beyond 5:1–7:1 did not lead to any noteworthy enhancement in yield. This plateau in yield might be attributed to the increased solvent usage at higher ratios, which in turn leads to higher costs, as noted by Balyan [21] *et al.* Therefore, for the purpose of optimization, the liquid-solid proportion was confined within the spectrum of 4:1–6:1.

Fig 1(B) depicts the consequences of particle size on the yields of CE, TPC, and TFC. The trends in yield for CE, TPC, and TFC were similar, reaching their maximum at a particle size of 850 µm. Both larger and smaller particle sizes resulted in diminished recoveries. This trend might be explained by the fact that a decrease in particle size allows for more thorough contact between the hempseed threshing residues powder and the extraction solvent, facilitating dissolution. However, a further reduction in particle size may lead to the powder becoming too fine, resulting in increased adsorption and agglomeration. This can heighten resistance to mass transfer and decrease the mass transfer rate, adversely affecting the dissolution of plant materials, as discussed by Pinelo [22] *et al.* Consequently, a particle size of 850 µm was identified as the optimum for extraction.

As depicted in Fig 1(C), the output of TPC, TFC, and CE augmented with longer extraction times. However, beyond 60 minutes, the increment in yield became less significant. Specifically, extraction times extending from 90 to 150 minutes resulted in reduced levels of phenolics and flavonoids. While a prolonged extraction duration can aid in the dispersion of target substances from the plant matrix into the solvent, as suggested by Pham [23] *et al.*, excessively long extraction periods, especially at elevated temperatures, may result in the breakdown of these bioactive substances [24]. Consequently, an extraction time range of 30–60 minutes was chosen for the optimization process.

The rise in ethanol concentration from 50% to 70% was associated with an increase in CE, TPC, and TFC, as demonstrated in Fig 1(D). However, a higher ethanol amount caused a notable reduction in the extraction of polyphenols and flavonoids. This pattern can be attributed to the fact that the polar hydroxyl groups in flavonoids and polyphenols require a polar solvent for effective extraction, as noted by Maria [25] *et al.*, Yet, a higher concentration of a strongly polar solvent might precipitate more impurities, hindering the dissolution of the target compounds, as described by Xie [26] *et al*. Additionally, the polarity of ethanol-water mixtures diminishes with increasing ethanol concentration. Hence, an ethanol content of 70% (v/v) was deemed optimal for extracting the highest levels of TPC and TFC from hempseed threshing residues. In this study, the ethanol concentration was selected to range from 60% to 80% for the optimization of TPC and TFC extraction.

### Optimization of extraction process by RSM

Table 1 presents the responses for CE, TPC, and TFC. The CE values varied from 3.69% (in run 19) to 4.79% (in run 28), TPC ranged between 18.51% (run 14) and 27.82% (run 5), and TFC spanned from 11.29% (run 4) to 16.69% (run 5). The ANOVA results, detailed in Table 2, show that the second-degree polynomial equations for CE, TPC, and TFC were statistically notable with *P*-values less than 0.0001. The *F*-values for these models were 117.67, 21.55, and 14.14, respectively, suggesting a mere 0.01% probability that these results could be attributed to random noise. The absence of fit was determined to be negligible for CE (0.69 > 0.05), TPC (1.4500 > 0.05), and TFC (0.6005 > 0.05), indicating a 71.02%, 38.57%, and 76.64% probability, respectively, that such a high lack-of-fit *F*-value could be a result of random variation. The *R*^2^ values for CE (0.9916), TPC (0.9556), and TFC (0.9339) indicated that the models explained 99.16%, 95.56%, and 93.39% of the variance in the responses, respectively. The Predicted *R*^2^ values for CE, TPC, and TFC were 0.9644, 0.7849, and 0.7304, respectively, aligning well with the Adjusted *R*^2^ values of 0.9831, 0.9113, and 0.8679. The Adeq Precision ratios of 35.531 for CE, 17.157 for TPC, and 12.969 for TFC suggest that these models can reliably navigate the design space. The regression equations (Eq. 5, Eq. 6, and Eq. 7) established the relationships between the four extraction factors and the responses for CE, TPC, and TFC, respectively.

**Table 1:** Box-Behnken Design and response values of TPC and TAC.

Run	Independent variables				Respond variables		
	Liquid-to-solid ratio X_1_ (mL/g)	Extraction time X_2_ (min)	Particle size X_3_ (µm)	Ethanol concentration X_4_ (%, v/v)	CE (%)	TPC (%)	TFC (%)
1	6 (1)	90 (1)	1210 (0)	70 (0)	4.28 ± 0.21	27.53 ± 1.28	14.79 ± 1.43
2	6 (1)	60 (0)	1210 (0)	60 (-1)	4.25 ± 0.43	24.15 ± 1.05	14.44 ± 1.64
3	5 (0)	60 (0)	2000 (1)	80 (1)	4.09 ± 0.26	23.12 ± 1.13	14.99 ± 1.64
4	5 (0)	30 (-1)	1210 (0)	60 (-1)	3.89 ± 0.30	18.62 ± 1.86	11.29 ± 1.89
5	5 (0)	60 (0)	1210 (0)	70 (0)	4.69 ± 0.25	27.82 ± 2.05	16.69 ± 1.43
6	4 (-1)	60 (0)	1210 (0)	80 (1)	4.21 ± 0.54	25.14 ± 1.96	15.11 ± 2.10
*7*	*5 (0)*	90 (1)	1210 (0)	80 (1)	*4.19 ± 0.32*	*25.53 ± 1.89*	*13.79 ± 1.96*
8	4 (-1)	60 (0)	420 (-1)	70 (0)	4.09 ± 0.76	25.24 ± 2.12	15.29 ± 1.59
9	6 (1)	30 (-1)	1210 (0)	70 (0)	3.91 ± 0.33	20.02 ± 2.08	11.83 ± 1.01
10	5 (0)	60 (0)	1210 (0)	70 (0)	4.75 ± 0.57	27.02 ± 2.25	15.42 ± 1.13
11	5 (0)	90 (1)	420 (-1)	70 (0)	4.18 ± 0.23	25.15 ± 1.07	15.52 ± 0.37
12	5 (0)	90 (1)	1210 (0)	60 (-1)	4.23 ± 0.26	25.07 ± 1.68	15.45 ± 0.53
13	5 (0)	60 (0)	420 (-1)	80 (1)	4.01 ± 0.32	22.16 ± 1.98	14.12 ± 1.65
*14*	*5 (0)*	30 (-1)	420 (-1)	*70 (0)*	*3.77 ± 0.27*	*18.51 ± 1.55*	*11.93 ± 0.55*
15	5 (0)	90 (1)	2000 (1)	70 (0)	4.08 ± 0.31	23.03 ± 1.76	13.91 ± 1.02
16	4 (-1)	90 (1)	1210 (0)	70 (0)	4.19 ± 0.53	23.01 ± 2.06	14.01 ± 1.42
17	6 (1)	60 (0)	2000 (1)	70 (0)	4.11 ± 0.53	24.53 ± 2.03	15.12 ± 0.75
18	6 (1)	60 (0)	1210 (0)	80 (1)	4.18 ± 0.32	24.73 ± 1.97	14.69 ± 0.97
19	5 (0)	30 (-1)	1210 (0)	80 (1)	3.68 ± 0.34	20.69 ± 1.86	14.27 ± 0.43
20	5 (0)	60 (0)	420 (-1)	60 (-1)	4.16 ± 0.40	22.07 ± 1.54	14.99 ± 1.09
21	5 (0)	60 (0)	2000 (1)	60 (-1)	4.01 ± 0.28	22.96 ± 1.78	13.84 ± 1.13
22	4 (-1)	60 (0)	2000 (1)	70 (0)	4.11 ± 0.27	24.14 ± 1.78	14.11 ± 0.96
23	4 (-1)	60 (0)	1210 (0)	60 (-1)	4.17 ± 0.19	22.31 ± 2.23	16.29 ± 1.84
24	4 (-1)	30 (-1)	1210 (0)	70 (0)	3.89 ± 0.25	22.88 ± 1.68	13.91 ± 1.42
25	5 (0)	60 (0)	1210 (0)	70 (0)	4.69 ± 0.28	26.13 ± 2.42	16.53 ± 0.86
26	5 (0)	30 (-1)	2000 (1)	70 (0)	3.77 ± 0.17	20.87 ± 2.17	13.03 ± 0.31
27	6 (1)	60 (0)	420 (-1)	70 (0)	4.18 ± 0.37	24.39 ± 2.84	14.28 ± 1.21
28	5 (0)	60 (0)	1210 (0)	70 (0)	4.79 ± 042	27.65 ± 1.97	15.72 ± 0.97
29	5 (0)	60 (0)	1210 (0)	70 (0)	4.71 ± 0.36	27.16 ± 1.54	15.68 ± 1.13

**Table 2:** Analysis of variance (ANOVA) for the quadratic polynomial model.

Source	CE					TPC					TFC				
	SS	DF	MS	F-value	p-value*	SS	DF	MS	F-value	p-value*	SS	DF	MS	F-value	p-value*
Model	2.4176	14	0.1727	117.67	4.95E-12	174.59	14	12.47	21.55	< 0.0001	45.0520	14	3.2180	14.14	6.59E-06
X_1_	0.0052	1	0.00527	3.55	0.0805	0.5764	1	0.5764	0.9959	0.3352	1.0620	1	1.0621	4.67	0.0486
X_2_	0.4181	1	0.41817	284.93	1.06E-10	64.0800	1	64.08	110.72	< 0.0001	10.472	1	10.472	46.01	8.83E-06
X_3_	0.0040	1	0.00407	2.748	0.1196	0.1064	1	0.1064	0.18	0.6746	0.1064	1	0.1064	0.47	0.5053
X_4_	0.0102	1	0.0103	6.96	0.0195	3.1900	1	3.19	5.52	0.0340	0.0374	1	0.0374	0.16	0.6913
X_1_X_2_	0.0012	1	0.0012	0.83	0.3764	13.6200	1	13.62	23.53	0.0003	2.0449	1	2.0449	8.98	0.0096
X_1_X_3_	0.0020	1	0.0020	1.38	0.2597	0.3844	1	0.3844	0.66	0.4287	1.0201	1	1.0201	4.482	0.0526
X_1_X_4_	0.0030	1	0.0030	2.06	0.1730	1.2700	1	1.27	2.19	0.1614	0.5112	1	0.5112	2.25	0.1561
X_2_X_3_	0.0025	1	0.0025	1.70	0.2129	5.0200	1	5.02	8.67	0.0107	1.8360	1	1.8360	8.07	0.0131
X_2_X_4_	0.0072	1	0.0072	4.92	0.0435	0.6480	1	0.648	1.12	0.3079	5.3824	1	5.3824	23.65	0.0003
X_3_X_4_	0.0132	1	0.0132	9.01	0.0095	0.0012	1	0.0012	0.0021	0.9640	1.0201	1	1.0201	4.48	0.0526
X_1_^2^	0.3572	1	0.3572	243.41	3.02E-10	3.4500	1	3.45	5.97	0.0284	1.4857	1	1.4857	6.53	0.02297
X_2_^2^	1.1629	1	1.1629	792.44	1.00E-13	53.6000	1	53.6	92.61	< 0.0001	19.776	1	19.776	86.89	2.21E-07
X_3_^2^	0.8449	1	0.8449	575.77	8.99E-13	31.8900	1	31.89	55.09	< 0.0001	4.3730	1	4.3730	19.21	0.0006
X_4_^2^	0.5728	1	0.5728	390.33	1.27E-11	30.5300	1	30.53	52.76	< 0.0001	1.9878	1	1.9878	8.73	0.0104
Residual	0.0205	14	0.0015			8.10000	14	0.5788			3.1863	14	0.2276		
Lack of Fit	0.0130	10	0.0013	0.69	0.7102	6.3500	10	0.6347	1.45	0.3857	1.91243	10	0.1912	0.6	0.7664
Pure Error	0.0075	4	0.0019			1.7600	4	0.439			1.2739	4	0.3185		
Cor Total	2.4381	28				182.690	28				48.2383	28			
R²	0.9916					0.9556					0.9339				
Adjusted R²	0.9831					0.9113					0.8679				
Predicted R²	0.9644					0.7849					0.7304				
Adeq Precision	35.5312					17.1573					12.9688				

P < 0.01 highly significant; 0.01 < P < 0.05 significant; P > 0.05 not significant.SS: sum of squares; DF: degree of freedom; MS: mean square.


$$\begin{array}{cc} & Y_{1}=-18.3123+2.5595X_{1}+0.0511X_{2}+0.0011X_{3}+0.4096X_{4}+0.0006X_{1}X_{2}-2.85E-\\ & 05X_{1}X_{3}-0.0028X_{1}X_{4}-1.05E-06X_{2}X_{3}+0.00014X_{2}X_{4}+7.28E-06X_{3}X_{4}-0.2347X_{1}^{2}-\\ & 0.000470463X_{2}^{2}-5.78E-07X_{3}^{2}-0.00297X_{4}^{2} \end{array}$$ (5)

$$\begin{array}{cc} & Y_{2}=-131.3136+7.2885X_{1}+0.3039X_{2}+0.0094X_{3}+3.4482X_{4}+0.0615X_{1}X_{2}+\\ & 0.00039X_{1}X_{3}-0.0563X_{1}X_{4}-4.73E-05X_{2}X_{3}-0.00134X_{2}X_{4}+2.22E-06X_{3}X_{4}-07297X_{1}^{2}-\\ & 0.0032X_{2}^{2}-3.55E-06X_{3}^{2}-0.0217X_{4}^{2} \end{array}$$ (6)

$$\begin{array}{cc} & Y_{3}=-21.9913-0.2176X_{1}+0.4500X_{2}-0.0029X_{3}+0.7565X_{4}+0.0238X_{1}X_{2}+\\ & 0.00064X_{1}X_{3}+0.0358X_{1}X_{4}-0.000029X_{2}X_{3}-0.0039X_{2}X_{4}+0.000064X_{3}X_{4}-\\ & 0.4786X_{1}^{2}-0.0019X_{2}^{2}-1.31563E-06X_{3}^{2}-0.0055X_{4}^{2} \end{array}$$ (7)

### Analysis of interaction between factors

In the statistical analysis, *P*-values are employed to determine the importance of model terms. Specifically, *P*-values less than 0.01 are indicative of highly significant model terms, those between 0.01 and 0.05 signify important model terms, and *P*-values higher than 0.05 are associated with terms that are not statistically significant. For CE, the model terms X_2_, the interaction term X_3_X_4_, and the square terms X_1_^2^, X_2_^2^, X_3_^2^, X_4_^2^ were statistically significant. The terms X_4_, and the interaction term X_2_X_4_ were significant, whereas the terms X_1_, X_3_, X_1_X_2_, X_1_X_3_, X_1_X_4_, and X_2_X_3_ were not statistically significant. In the case of TPC, X_2_, the interaction term X_1_X_2_, and the square terms X_2_^2^, X_3_^2^, and X_4_^2^ emerged as highly significant. The terms X_1_, the interaction term X_2_X_3_, and the square terms X_1_^2^, X_4_^2^ were significant. The terms X_3_, X_1_X_3_, X_1_X_4_, X_2_X_4_, and X_3_X_4_ were not significant. For TFC, X_2_, the interaction factors X_1_X_2_ and X_2_X_4_, and the quadratic terms X_2_^2^ and X_3_^2^ were highly significant. The terms X_1_, the interaction term X_2_X_3_, and the square terms X_1_^2^ and X_4_^2^ were significant. The terms X_3_, X_4_, X_1_X_3_, X_1_X_4_, and X_3_X_4_ were not significant.

**Fig 2:**
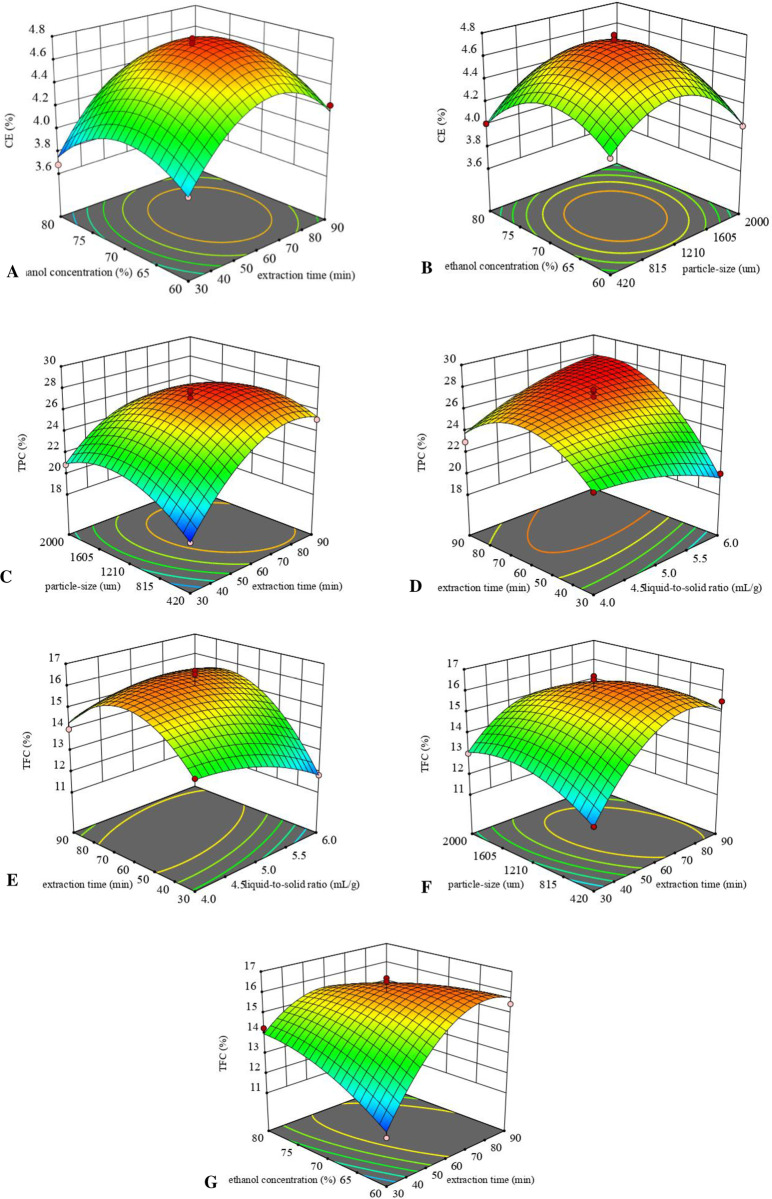
Response surface parameter for the CE, TPC and TFC. (A) extraction time and ethanol concentration for the CE; (B) particle size and ethanol concentration for the CE; Response surface parameter for the TPC: (C) liquid-solid proportion and extraction time for the TPC; (D) extraction time and particle size for the TPC; (E) liquid-solid proportion and extraction time for the TFC; (F) extraction time and particle size for the TFC; (G) extraction time and ethanol concentration for the TFC.

Fig 2 displayed the response surface plots for CE, TPC, and TFC, respectively. In Fig. 2(A), the quadratic relationship between extraction time and ethanol concentration on CE yield is depicted, holding the liquid-solid proportion at 5:1 and particle dimensions constant at 1210 µm. An upward trend in CE yield is observed as extraction time approaches 66.63 minutes and ethanol concentration reaches 69.61%, beyond which a decrease in yield is noted. Fig 2(B) illustrates that CE yield increases with the augmentation of both particle size and ethanol concentration, peaking at a particle size of 1180.14 µm and an ethanol concentration of 69.61%. Here, isolating duration and ethanol content are demonstrated to have a significant impact on CE yield. The reason could be that prolonged extraction time allows for more comprehensive destruction of plant tissue cells, enhancing the solubility of crude extracts. However, an extended period of high temperature, light, or oxygen exposure during extraction can lead to the decomposition of some active ingredients, as noted by Naczk [27] *et al.*

Fig 2(C) demonstrates the consequences of the liquid-solid proportion and extraction duration on the extraction output. The optimal extraction output was achieved with a liquid-solid proportion of 6.00:1 and an isolating duration of 82.43 minutes. It was noted that an elevation in the liquid-solid proportion as well as isolating duration initially led to an increase in TPC yield, but further increases led to a reduction in output. Fig 2(D) presents the impacts of extraction duration and particle size on TPC. A rise in both processing time and particle size initially contributed to a higher TPC yield. However, excessively large increases in these parameters led to reduced extraction yields. The interplay between extraction duration and particle dimensions displayed both linear and interaction effects on the output. The maximum TPC production was noted at an extraction duration of 82.43 minutes and a particle dimension of 1131.67 µm. This indicates the necessity of balancing these parameters to optimize extraction efficiency.

Fig 2(E) displays the consequences of the liquid-solid proportion and extraction duration on the TFC output, maintaining a particle size of 1210 µm and an ethanol concentration of 70%. It was observed that TFC yield escalated with increases in both extraction duration and liquid-solid proportion. Notably, extraction duration exerted a more pronounced impact on TFC output than the liquid-solid proportion. A rapid enhancement in TFC output occurred as extraction time extended from 30 minutes to 78.76 minutes, followed by a swift decline when the extraction time ranged from 78.76 minutes to 90 minutes. In Fig 2(F), the correlation between extraction duration and particle size on TFC output is showcased. The peak TFC yield was recorded at an extraction duration of 78.76 minutes and a particle dimension of 559.48 µm. The findings indicate that TFC yield benefits from an increase in both particle size and extraction duration. Fig 2(G) highlights the significant interactions between extraction duration and ethanol concentration on TFC output. An upward trend in yield was seen with increasing extraction time, up to 78.76 minutes. However, beyond this extraction time and with increasing ethanol concentration, there was a noticeable decline in TFC yield. This trend underscores the importance of carefully balancing extraction duration and ethanol content to maximize TFC extraction.

### Optimization of extraction parameters and model validation

In accordance with the three quadratic polynomial equations, it was evident that different factors exerted varying degrees of influence on each response value. To enhance the outputs of CE, TPC, and TFC, a comprehensive consideration of these three response indices was necessary. The BBD-RSM was employed to optimize the quadratic regression model for these indicators. The ideal extraction circumstances were determined to be: a liquid-solid proportion of 5.12:1, an extraction time of 69.71 minutes, a particle size of 1150.50 µm, and ethanol amount of 69.59% (v/v). In these circumstances, the anticipated values for CE, TPC, and TFC were 4.74%, 27.68%, and 16.14%, respectively. However, considering practical operational feasibility, these parameters were slightly adjusted to a liquid-solid proportion of 5.12:1, an extraction time of 70 minutes, a particle size of 1150 µm, and an ethanol content of 70% (v/v). To validate the accuracy of the model, three replicate tests were performed using these optimized settings. The real yields obtained for CE, TPC, and TFC were 4.67%, 27.54%, and 16.02%, respectively. These experimental results were in alignment with the anticipated values (*p* > 0.05), showing the dependability of the optimized model. Therefore, the parameters derived from the optimization can be confidently used for the extraction of CE, TPC, and TFC.

RSM proved to be an effective approach for integration with heat reflux extraction technologies, optimizing the output of total phenolic and flavonoid compounds from hempseed threshing residues. Typically, a higher liquid-solid proportion and a smaller particle dimension can enhance the mass transfer driving force [22,28]. Meanwhile, an optimal extraction time and ethanol concentration can improve the solubilization of target substances in the isolating solution [11,29,30]. Consequently, traditional heat reflux extraction emerged as a potent technique for extracting compounds with high bioactivity [31]. Moreover, the high consistency between experimental and predicted results underscored the reliability of the approach. Hence, the three quadratic polynomial models developed through a three-level, four-factor BBD were validated as accurate for guiding the experimental scheme [32]. This alignment confirms the credibility and applicability of these models in optimizing extraction processes.

### Analysis of chemical compounds in the extracts

**Fig 3:**
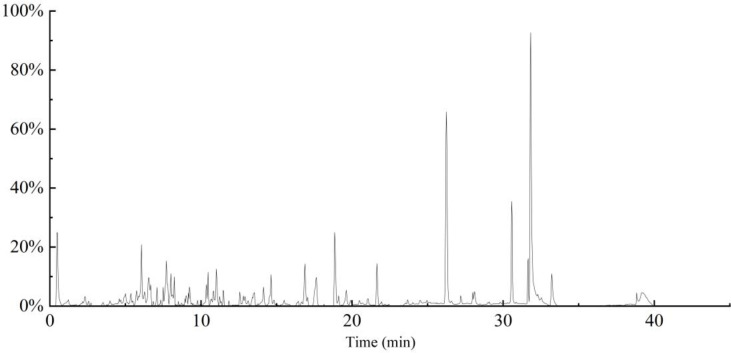
TIC of the extracts by HPLC- MS.

The fingerprinting of ion total chromatogram (TIC) of the extracts by HPLC- MS is as Fig 3 and the majority of compounds in the hempseed threshing residues extracts are identified in the Table 3. Most of the compounds found may belong to the class of polyphenol and flavonoids. The most common polyphenolic compounds found in the hempseed threshing residues extracts may be myricitrin, tetramethoxy-trans-stilbene, cannabisin A, and tri-p-coumaroylspermidine. Quercetin-3-O-arabinose, kuromanin, 3’3-demethyl-grossamide, vitexin-2-O-rhamnoside, and glucosylvitexin may be the most common flavonoids compounds [33]. In addition, the results showed high levels of the class of terpenes (peltatoside E, methyltoosendanpentol, 14(R)-Hydroxy-7β-isovaleroyloxyoplop-8(10)-en-2-one), lignans (pinoresinol diglucoside) and alkaloids (Stemospironine) [34].

**Table 3:** Identification of the extracts by HPLC-MS in negative mode.

Peak NO.	Compound Name	Molecular Formula	Retention Time (min)	Experimental Mass	Intensity	Error (ppm)
1	Hydroxymethylfurfural acid	C_6_H_6_O_4_	2.33	143.0336	18604	-1.7
2	Ginkgolide-J	C_20_H_24_O_10_	5.00	425.1415	5084	0.5
3	Peltatoside E	C_16_H_28_O_8_	5.74	349.1830	14424	-1.9
4	Curcolone	C_15_H_18_O_3_	6.06	247.1323	5087	-1.0
5	Asplenetin-2-O-D-glucoside	C_21_H_30_O_8_	6.29	411.1984	13518	-1.4
6	Mulberrofuran G	C_25_H_28_O_8_	6.51	562.5792	18048	-0.7
7	Trans-Ferulic aldehyde	C_10_H_10_O_3_	6.56	179.0698	11060	0.4
8	Phelligenin	C_15_H_10_O_7_	7.06	303.0494	18250	0.3
9	3,5,7,2’,6’-Pentahydroxyflavone	C_15_H_12_O_7_	7.62	305.0649	11230	-1.2
10	Norplatanin	C_13_H_14_O_9_	7.67	315.1711	12236	0.3
11	Myricitrin	C_21_H_20_O_12_	7.72	463.1032	60713	0.9
12	Quercetin-3-O-arabinose	C_20_H_18_O_11_	8.01	435.0920	31137	-0.4
13	Kuromanin	C_21_H_20_O_11_	8.23	449.1073	27701	-1.1
14	Diethyl piscidate	C_15_H_20_O_7_	9.04	313.1255	8920	-8.4
15	10-Pinenyl vicine glycoside	C_21_H_34_O_10_	9.19	447.2196	9576	1.7
16	Polygalae D	C_18_H_24_O_9_	9.26	385.1467	21380	0.3
17	Abietyl acetate	C_20_H_24_O_6_	11.01	361.1619	8966	-2.5
18	3-O-Acetyl-2-O-(3-methoay-4-hydrozycinnamoyl)-α-L-rhamnose	C_18_H_22_O_9_	11.25	381.1316	16548	1.3
19	Ganodermic acid S	C_30_H_44_O_3_	13.48	453.3361	18992	-0.6
20	(-)-Olivil 4’‘-O-beta-D-glucopyranoside	V_26_H_34_O_12_	14.14	539.2097	21388	-0.8
21	Stemospironine	C_19_H_29_NO_5_	14.64	352.2090	11523	-8.2
22	Tetramethoxy-trans-stilbene	C_18_H_20_O_4_	17.63	301.1411	89070	-7.7
23	Coronaric acid	C_18_H_32_O_3_	18.85	297.2398	183659	2.3
24	Cannabisin A	C_34_H_30_N_2_O_8_	21.64	594.2002	103727	0.4
25	14(R)-Hydroxy-7β-isovaleroyloxyoplop-8(10)-en-2-one	C_20_H_32_O_4_	23.68	337.2344	17933	-1.7
26	Pinoresinol diglucoside	C_32_H_42_O_16_	26.21	681.2394	216228	0.6
27	Di-(2-ethylhexyl) phthalate	C_24_H_38_O_4_	26.28	391.2838	2377	-1.4
28	Methyltoosendanpentol	C_31_H_52_O_6_	28.11	521.3806	20472	-6.0
29	3’3-Demethyl grossamide	C_34_H_32_N_2_O_8_	30.55	596.2159	73958	1.28
30	Tri-p-coumaroylspermidine	C_34_H_37_N_3_O_6_	31.82	583.2688	173748	0.51
31	Vitexin-2-*O*- rhamnoside	C_27_H_30_O_14_	33.22	577.1557	60236	-1.2
32	Glucosylvitexin	C_27_H_30_O_15_	38.83	593.1511	20363	-0.9

### Analysis of antioxidant activity

**Fig 4:**
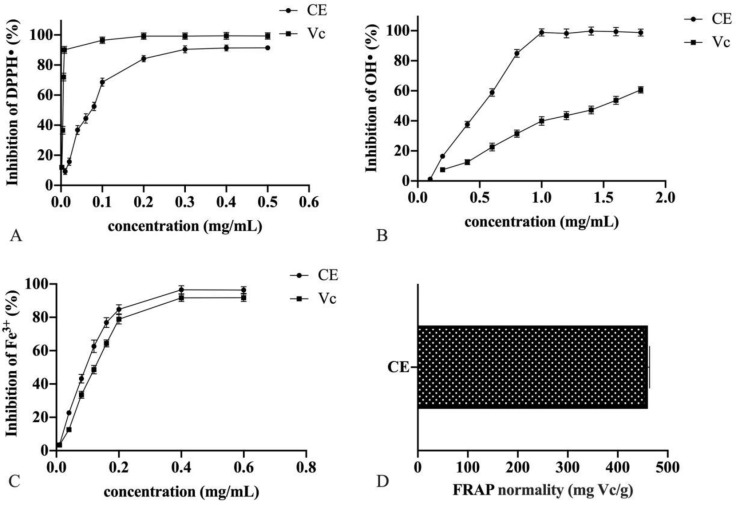
Scavenging activity of the crude extract and Vc against DPPH.· (A), ·OH (B) radicals, Fe^3+^ (C) and FRAP (D).

In this research, the antioxidative capacities of CE from hempseed threshing residues were evaluated using various assays, including DPPH· scavenging,·OH scavenging, Fe^3+^ scavenging, and FRAP assays (Fig 4A-D). Fig 4A shows that CE exhibited concentration-dependent scavenging capabilities on DPPH· radicals within a span of concentrations ranging from 0.01–0.20 mg/mL. The scavenging capabilities stabilized at CE concentrations exceeding 0.02 mg/mL. The IC_50_ value for CE was determined to be 0.065 mg/mL, 13 times greater than that of Vc at 0.005 mg/mL. Notably, hempseed threshing residue extracts, when prepared utilizing various solvent combinations, demonstrated superior scavenging activities (IC_50_ approximately 90–2210 μg/mL for DPPH radicals) compared to extracts from hemp kernels (IC_50_ approximately 580–5630 μg/mL) as documented by Chen [35] *et al*. This finding suggests that hempseed threshing residues, typically disposed of as waste, can be a promising origin of antioxidants [36]. The·OH scavenging activity of CE (Fig 4B) followed a similar pattern to the DPPH· scavenging activity. CE showed significantly higher scavenging ability (*p* < 0.05) at a level of 1.0 mg/mL. Beyond this concentration, the scavenging potential plateaued, with an IC_50_ of 0.409 mg/mL. In contrast, Vc, utilized as a positive reference, demonstrated a linear scavenging pattern within the range of 0.1–1.8 mg/mL and had an IC_50_ of 1.439 mg/mL, threefold higher than CE. This indicates that CE has a stronger scavenging potential compared to Vc. Regarding Fe^3+^ scavenging (Fig 4C), CE effectively inhibited Fe^3+^ in a significant, concentration-associated way within the span of concentrations ranging from 0.01–0.6 mg/mL. The IC_50_ of CE (0.075 mg/mL) was slightly less than that of Vc (0.101 mg/mL), indicating that CE and Vc are comparably effective. The reducing power of CE, a key indicator of potential antioxidant activity, was also assessed and expressed as Vitamin C equivalents (mg Vc/g), shown in Fig. 4D. The reductive ability of CE was found to be 460.36 mg Vc/g, highlighting its substantial antioxidant capacity.

### Cell viability

**Fig 5:**
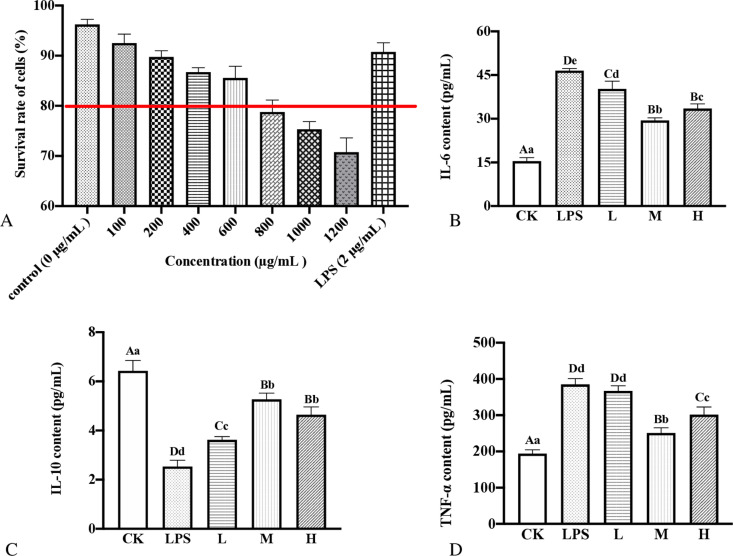
Effects of extracts on inflammatory factors production in LPS-induced RAW264.7 cells. (A) the cell viability was determined by MTT method, (B) Secretion of interleukin (IL)-6, (C) Secretion of interleukin (IL)-10, (D) Secretion of interleukin TNF-α. Data were presented as means ± SD (n = 3). The difference of capital letters of A and B represented significant difference (P < 0.01); Different lowercase letters of a, b and c represent significant differences (P < 0.05).

Fig 5(A) illustrates a notable decline in cell survival rates with rising concentrations of hempseed threshing residue extracts. Concentrations ranging from 100–600 μg/mL of these extracts, alongside 2 μg/mL of LPS, exhibited minimal impact on the development of RAW.264.7 cells, maintaining over 80% viability. Consequently, levels of 100 μg/mL (low dose), 200 μg/mL (medium dose), and 400 μg/mL (high dose) were chosen for further experiments with hempseed threshing residue extracts.

### Analysis of IL-6, IL-10 and TNF-α

Macrophages are known to rapidly release various interleukins and tumor necrosis factors, influencing the progression of inflammatory processes [20,37]. Accordingly, the concentrations of IL-6, IL-10, and TNF-α secretion were used to assess the immunomodulatory properties of hempseed threshing residue extracts. The impact of these extracts on the IL-6, IL-10, and TNF-α levels in RAW264.7 cells is depicted in Fig 5(B-D). The data indicate that the extracts alleviate LPS-induced inflammation in RAW264.7 cells. As illustrated in Fig. 5(B), IL-6 levels were significantly reduced across all dosage groups (low, medium, and high) in comparison to the LPS-only group (*P* < 0.05), though no notable discrepancy was noted between the medium and high dosage groups (*P* < 0.01). Fig 5(C) reveals a substantial increase in IL-10 levels in all extract-treated categories in contrast to the LPS category (*P* < 0.01), with no noteworthy distinction between the medium and high dosage groups (*P* < 0.05). Fig 5(D) demonstrates that TNF-α levels significantly decreased in both medium and high dosage groups (*P* < 0.01), however, there was no significant distinction noted between the low-dosage group and the LPS group (*P* < 0.01). Consequently, the 200 μg/mL concentration of hempseed threshing residue extracts exhibited optimal anti-inflammatory efficacy. Cho [38] *et al.* also observed similar anti-inflammatory effects in their study on Pepper flavonoids, noting a reduction in pro-inflammatory agents in LPS-activated RAW 264.7 cells. Additionally, Cao [39] *et al.* reported that *G. procumbens* flower extracts suppressed IL-6, TNF-α, and their mRNA transcriptions in LPS-activated RAW264.7 macrophage cells. Industrial hemp leaves extracts reduced LPS-induced inflammation in RAW 264.7 Cells and reduced IL-1β-stimulated production of inflammatory mediators in human chondrocytes [40]. Martinez [41] *et al.* identified amounts of phenolic compounds which significantly reduced the inflammatory competence of LPS-treated human primary monocytes, decreasing TNF-α and IL-6 gene expression and secretion. These outcomes are consistent with the present research, suggesting that the inflammation inhibitory action of hempseed threshing residue extracts may be because of the existence of total polyphenols and flavonoids content.

## Conclusions

In this investigation, the BBD of RSM was utilized to improve the extraction of polyphenols and flavonoids from hempseed threshing residues, resulting in a noteworthy rise in the output of crude extract. The optimal parameters for the extraction process, including liquid-solid proportion, extraction duration, particle size, and ethanol content, were established to maximize the CE yield, TPC, and TFC. Under these optimal parameters, the outputs of CE, TPC, and TAC were found to be 4.67%, 27.54%, and 16.02%, respectively. The experimental results closely matched the predicted outcomes, demonstrating the reliability and accuracy of the BBD models and the polynomial regression model. Additionally, the extracts derived from hempseed threshing residues exhibited significant antioxidant properties and strong immunomodulatory activity. These findings highlight the potential of utilizing hempseed threshing residues in food or health product industries as natural alternatives to synthetic antioxidants. However, the antioxidant and immune activity of hempseed threshing residues extracts under physiological conditions in vivo, and the relevant mechanism should be further studied. Future research is recommended to explore the anti-tumor properties and broader health benefits of these extracts for potential applications in the pharmaceutical sector.
